# Combined stimuli of cold, hypoxia, and dehydration status on body temperature in rats: a pilot study with practical implications for humans

**DOI:** 10.1186/s13104-020-05375-w

**Published:** 2020-11-11

**Authors:** Tadashi Uno, Tatsuya Hasegawa, Masahiro Horiuchi

**Affiliations:** Division of Human Environmental Science, Mount Fuji Research Institute, Kami-yoshida 5597-1, Fuji-yoshida-city, Yamanashi 4030005 Japan

**Keywords:** Area under the curve, Heat loss index, High-altitude, Hypothermia, Metabolism, Set point

## Abstract

**Objective:**

As human thermoregulatory responses to maintain core body temperature (T_core_) under multiple stressors such as cold, hypoxia, and dehydration (e.g., exposure to high-altitude) are varied, the combined effects of cold, hypoxia, and dehydration status on T_core_ in rats were investigated. The following environmental conditions were constructed: (1) thermoneutral (24 °C) or cold (10 °C), (2) normoxia (21% O_2_) or hypoxia (12% O_2_), and (3) euhydration or dehydration (48 h water deprivation), resulted in eight environmental conditions [2 ambient temperatures (T_a_) × 2 oxygen levels × 2 hydration statuses)]. Each condition lasted for 24 h.

**Results:**

Normoxic conditions irrespective of hypoxia or dehydration did not strongly decrease the area under the curve (AUC) in T_core_ during the 24 period, whereas, hypoxic conditions caused greater decreases in the AUC in T_core_, which was accentuated with cold and dehydration (T_a_ × O_2_ × hydration, *P* = 0.040 by three-way ANOVA). In contrast, multiple stressors (T_a_ × O_2_ × hydration or T_a_ × O_2_ or O_2_ × hydration or T_a_ × hydration) did not affect locomotor activity counts (all *P* > 0.05), but a significant simple main effect for O_2_ and T_a_ was observed (*P* < 0.001). Heat loss index was not affected by all environmental conditions (all *P* > 0.05). In conclusion, decreases in T_core_ were most affected by multiple environmental stressors such as cold, hypoxia, and dehydration.

## Introduction

Most of mammal’s (including human) energy regulates core body temperature (T_core_). Various environmental factors such as heat, cold, hypoxia, humidity, or wind would affect T_core_. Although physiological adaptation to environmental stressors is often studied in isolation, these stressors are frequently combined outside of laboratory settings. At high altitudes, both barometric pressure and ambient temperature (T_a_) decreases with an increase in altitude.

Cold exposure reduces human cutaneous blood flow that decreases heat transfer from the core to peripheral tissues [1]. This decreases in skin temperature, which narrows the gradient for cutaneous heat loss, and promotes heat conservation, and is vital to the prevention of hypothermia in cold. Contrariwise, hypoxic conditions reduce metabolism and core body temperature (T_core_) in many small mammals [2], primates [3], and humans [4–7]. In humans, this may be explained by peripheral circulation. Simulated high altitude elicits a cutaneous hyperemia that is mediated at the tissue level [8, 9], suggesting an acceleration in the rate of core cooling. The combined effects of cold and hypoxia may have competing effects on cutaneous circulation, and hence, T_core_ changes would be complicated. There are several issues to consider when investigating the combined effects of cold and hypoxia on T_core_ and/or peripheral circulation in humans. Previous human studies of cold and hypoxia have been conducted in a relatively short period (within 2 h) [9–13]. Generally, when humans are exposed to high-altitude such as climbing a mountain, exposure of several hours or a few days can occur. Thus, it may be difficult to expose people to multiple environmental stressors for a longer time due to ethical problems and strain on the participants. Importantly, greater individual variances in response to multiple stressors, i.e., cold and hypoxia, exist in humans [13]. Specifically, changes in T_core_ under exposure to hypoxia in cold stress cannot be explained by only peripheral circulation [13]. An initial step using animal modes (considered to have less individual variance), is required.

As heat dissipation caused by fluid redistribution is important in thermoregulation, the hydrations status on T_core_ should also be considered. For example, hyperventilation-induced dehydration is observed at high-altitude [14]. Similarly, inhalation of hypoxic gas causes an increase in urine volume, also suggesting dehydration status [15]. However, little is known about the combined effects of cold, hypoxia, and hypohydration on T_core_ irrespective of whether in humans or animals.

Accordingly, the main aim of this pilot study was to investigate the combined effect of cold, hypoxia, and dehydration on T_core_ and related factors using animal models. We hypothesized that multiple stimuli (cold, hypoxia, and dehydration) would cause the greatest reductions in T_core_.

## Main text

### Methods

#### Animals

Experiments were performed using age-matched (10–14 weeks), male, Wistar rats weighing 250–320 g (n = 40). Animals were maintained in a temperature-controlled ambient temperature (T_a_ = 24 °C) and relative humidity (50%), fed ad libitum, and kept on a 12 h light–dark cycle. All experiments were performed in accordance with the Ethics Committee for Animal Experiments, Mount Fuji Research Institute, Yamanashi Prefecture Government (ECAE-03–2016).

#### Experimental procedures

**Fig. 1:**
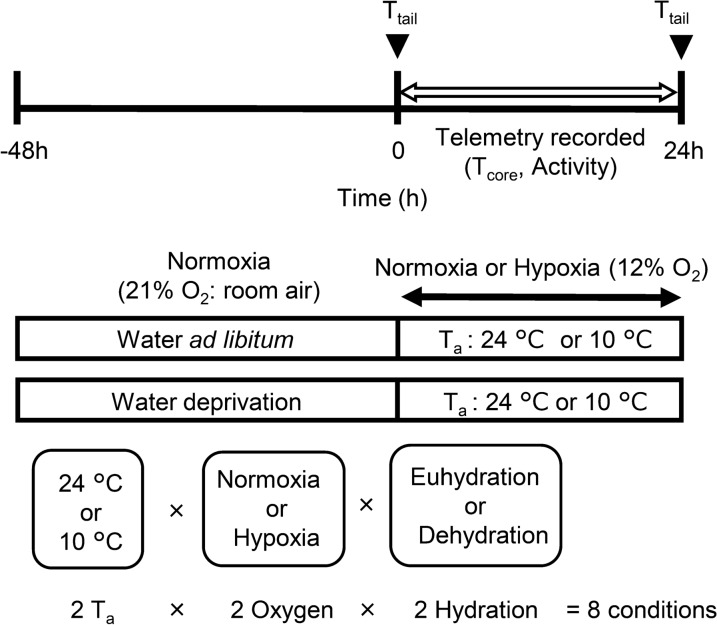
Experimental protocols. T_core_, core temperature; T_tail_, skin temperature of the tail; T_a_, ambient temperature

Environmental conditions were as follows; (1) thermoneutral (24 °C T_a_) or cold (10 °C T_a_), (2) normoxia (21%O_2_; room air) or normobaric hypoxia (12%O_2_), (3) euhydration (48 h ad libitum access to water before the experiment) or dehydration (48 h water deprivation before the experiment) (Fig. 1). In previous studies, 5 °C T_a_ [16, 17] or 10 °C T_a_ [18], and 10%O_2_ [16, 19, 20] or 7–10–12%O_2_ [21] were used. In our preliminary experiments, a few rats fell into asphyxia condition under 5 °C, therefore, we conducted the experiment of 10 °C T_a_. Regarding to O_2_ concentration, O_2_ saturation acutely decrease around below 50–60 torr PO_2_, at which is almost equivalent to 12%O_2_, based on oxygen–hemoglobin dissociation curve [22]. To produce dehydration status, 48 h water deprivation was enforced before the experiment, and the experiment lasted 24 h, resulted in 72 h water deprivation [23]. Eight experiments (2 T_a_ × 2 oxygen × 2 hydration status = 8 conditions; n = 5 for each) were performed. Ambient temperature (24 °C or 10 °C) was maintained in a climatic chamber (MIR-153; SANYO Electric Co., Ltd, Japan). The oxygen concentrations in the vinyl tent surrounding the climatic chamber were set at normobaric normoxia (21%O_2_) or hypoxia (12%O_2_). Hypoxic gas (12%O_2_) was supplied via a generator (Hypoxico Everest Summit II; Will Co., Ltd., Tokyo, Japan) and oxygen concentration was verified before and after each experiment (AE-300; Minato Medical Science, Osaka, Japan). The rats were weighed before and after the experiment.

#### General surgical preparation

Before the experiment, animals were anesthetized with an anesthetic mixture of medetomidine, midazolam, and butorphanol. For the measurements of T_core_, a radio transmitter (15 × 30 × 8 mm; Physio Tel TA10TA-F40, Data Sciences International Co., Ltd., St. Paul, MN, USA) was placed in the abdominal cavity of each rat. The rats were allowed to recover for at least one week before measurements. After each experiment, a radio transmitter was taken under anesthesia, thereafter, exsanguination from a cut through the heart aorta was applied for euthanization.

#### Measurements

In addition to the measurement of T_core_, infrared thermography (Thermo Shot F30; NEC Avio Infrared Technologies Co., Ltd., Japan) was used to measure tail skin temperature (T_tail_). Counts of locomotor activity (an indicator of behavior) were recorded every minute with a data collection system, which consisted of a receiver board (model RLA2000, Data Sciences International, Co., LTD, St. Paul, MN, USA) under the cage connected to a personal computer. The locomotor activity counts reflected positional movements but did not show other movements such as grooming or food intake.

#### Data analysis

The area under the curve (AUC; 0–24 h) of T_core_ was calculated from values measured at 0 h, using data every 1 min and the trapezoidal method. T_tail_ was the averaged values from the first and second thirds of the tail. Heat Loss Index (HLI) as an indicator of peripheral vasomotor activity, was calculated using the following equation:

$$Heat\; Loss \;Index\; (HLI)=\frac{({T}_{tail}-{T}_{a})}{({T}_{core}-{T}_{a})}$$

The value of HLI ranges from 0 (full vasoconstriction) to 1 (full vasodilation).

Changes in HLI (ΔHLI) were calculated by the difference between pre (time = 0 h)- and post (time = 24 h)- exposure to each environmental condition [24].

#### Statistics

Values are represented as mean ± standard deviation. All statistics were performed using a R software (ver. 3.1.3). A three-way ANOVA (T_a_ × O_2_ × Hydration) was performed for comparisons of the AUC, HLI, and activity counts. If significant *F* values were obtained, Bonferroni’s *post-hoc* test was used for further comparisons. A *P* value < 0.05 was defined as statistically significant.

### Results

Dehydration status (irrespective of T_a_ or O_2_ conditions) decreased body weight compared with the control conditions (24 °C T_a_, 21%O_2_, and euhydration) (F = 95.99, *P* < 0.001] (Additional file 1: Table S1). Conversely, hypoxia or cold per se did not affect body weight changes.

**Fig. 2:**
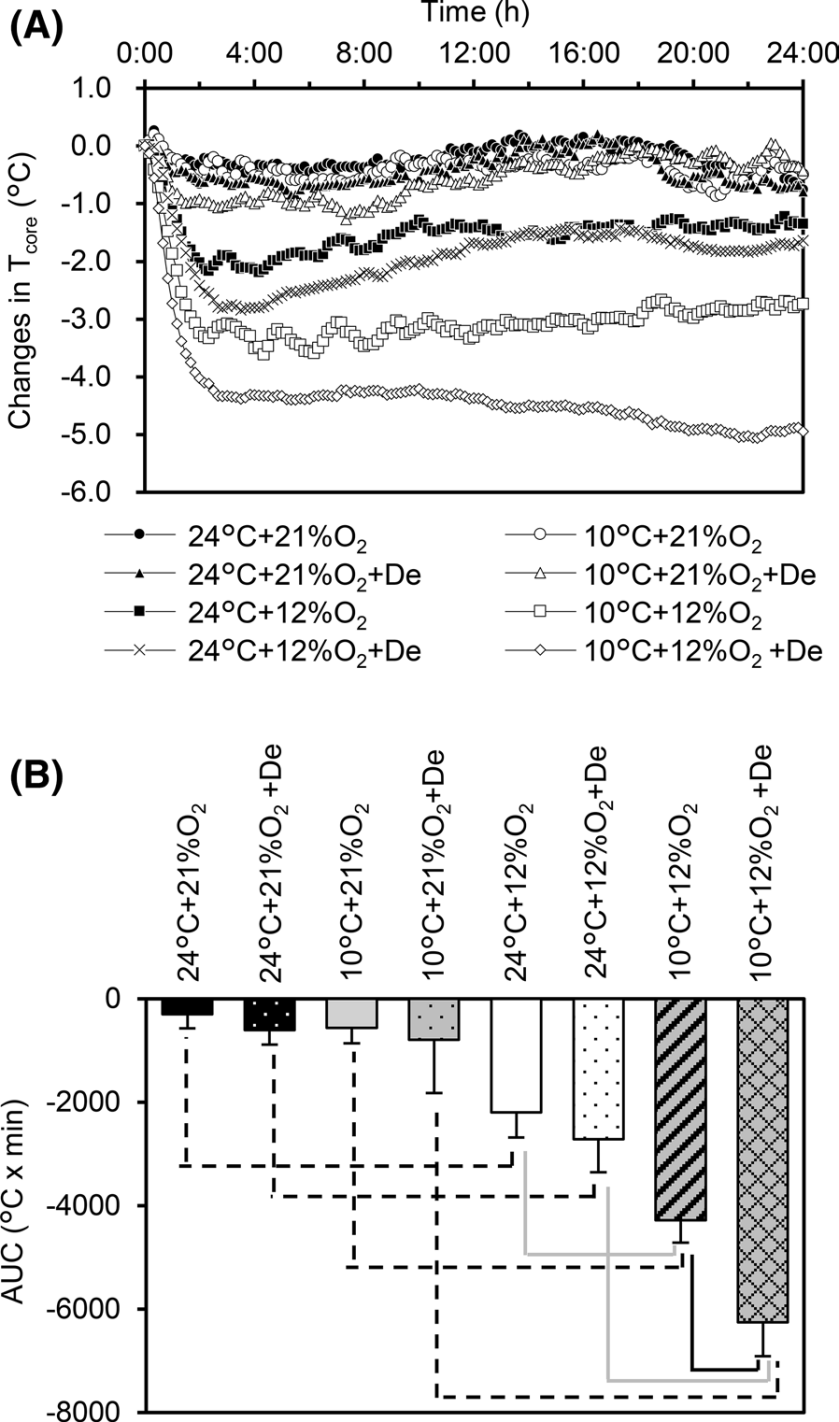
Time course of T_core_ among different environmental conditions. Values are given as only means (n = 5 for each, **a**). Mean values of the area under the curve (AUC) with standard deviation (SD) among all conditions throughout the 24 h experimental period (**b**). Hypoxia significantly decreased the AUC irrespective of cold and dehydration (dashed black lines). In hypoxia, cold environment (10 °C) further decreased the AUC in both euhydration and dehydration conditions (solid gray lines). Dehydration affected the AUC only in both hypoxia and cold (solid black line)

**Fig. 3:**
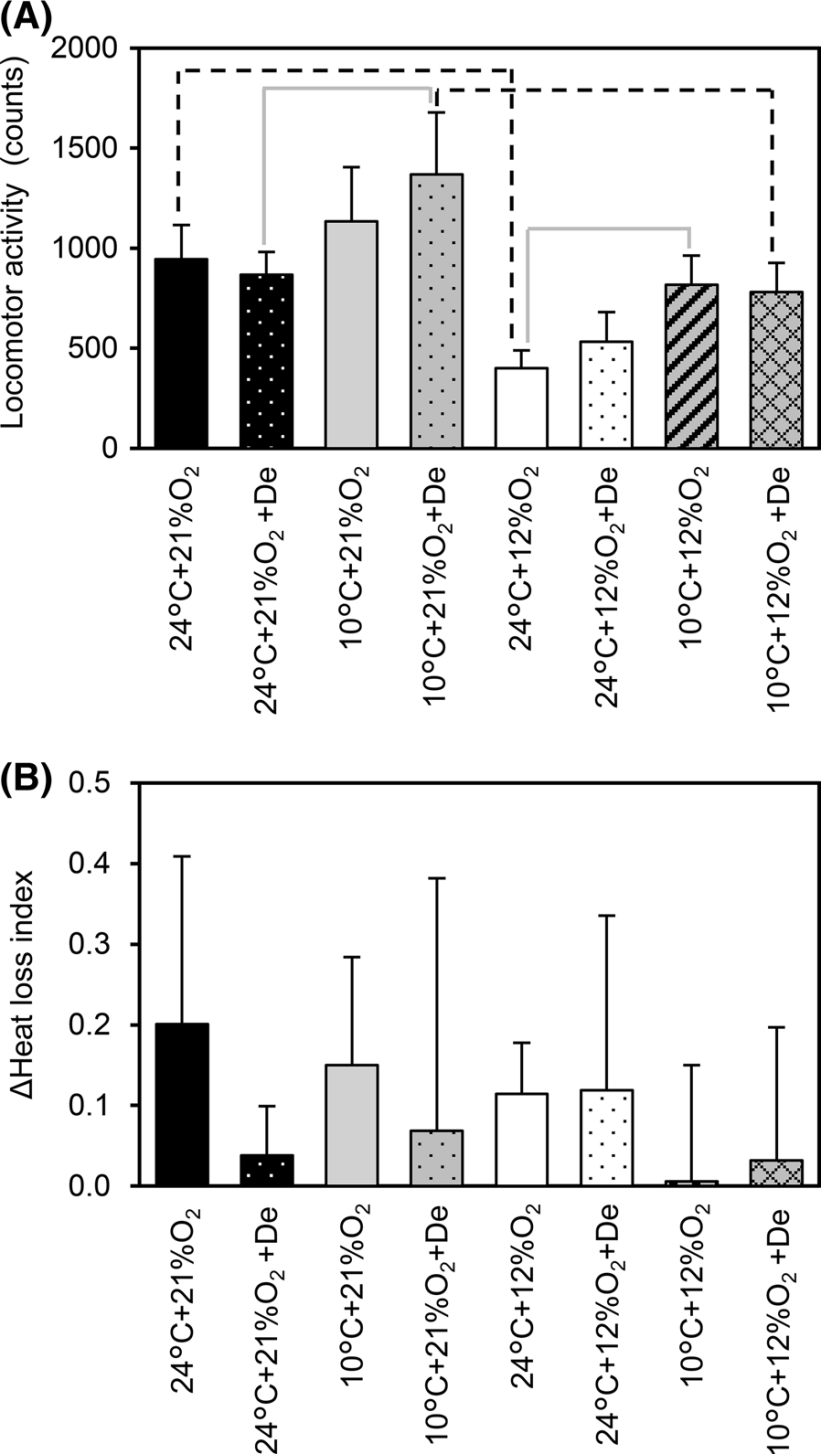
Mean values of the locomotor activity with SD among all conditions throughout the 24 h experimental period (**a**). Hypoxia significantly decreased the locomotor activity irrespective of cold and dehydration (dashed black lines). Dashed black and solid gray lines indicate significant differences when considering a simple main effect for oxygen and T_a_. Mean values of the heat loss index with SD among all conditions throughout the 24 h experimental period (**b**)

Mean values of T_core_ in each condition and comparisons of the T_core_ AUC are shown in Fig. 2. To avoid difficulties of observation in time course changes in T_core_, mean values without SD are shown (Fig. 2a). Normoxic conditions (irrespective of hypoxia or dehydration) did not decrease the AUC, whereas, hypoxic conditions caused greater decreases in the AUC, and were accentuated with cold and dehydration. A second-order interaction (T_a_ × O_2_ × hydration) was observed by three-way ANOVA (F = 4.570, *P* = 0.040, Fig. 2b). Figure 3a shows comparisons of activity in each condition. A three-way ANOVA revealed a trend in a second-order interaction (T_a_ × O_2_ × hydration; F = 4.066, *P* = 0.052), while no significant simple interactions were observed (T_a_ × O_2_; F = 0.011, *P* = 0.918, O_2_ × hydration; F = 0.065, *P* = 0.800, T_a_ × hydration; F = 0.357, *P* = 0.554). A simple main effect of O_2_ and T_a_ was observed (T_a_; F = 32.30, O_2_; F = 56.202, *P* < 0.001). Comparisons in HLI under all conditions showed no significant differences in the simple main effect, a simple interaction, and second order interaction were observed (all *P* > 0.05) (Fig. 3b).

### Discussion

The major findings of this pilot study were two-fold: (1) the AUC of T_core_ was mostly affected by multiple stressors (cold, hypoxia, and dehydration), (2) activity counts and HLI were not affected by multiple stressors.

The T_core_ decreased slightly within a few hours in normoxia (about − 0.1 to − 0.5 °C decrease per hour); however, these initial reductions in the T_core_ were markedly greater under hypoxia (about − 1 to − 2.5 °C decrease per hour). During acute exposure to a cold environment, shivering thermogenesis is activated to augment heat production [18, 25, 26], compensating for the increase in heat loss that occurs at low T_a_. In rats, shivering thermogenesis is gradually replaced by non-shivering thermogenesis during cold acclimation (four weeks) [27], which allows the animal to maintain high body temperatures under low T_a_ conditions. In the present experiment, as exposure time was 24 h, both shivering and non-shivering thermogenesis may have contributed to maintain T_core_ during the 24 h period under normoxic conditions, irrespective of T_a_ and dehydration, although the precise mechanisms are unclear. In hypoxia, a similar initial decrease in T_core_ has been observed [20, 28], and it is generally a consequence of a decrease in the T_core_ set-points, known as hypoxia-induced anapyrexia [29–31]. Therefore, these greater reductions in set-point in hypoxia may be associated with non-recovery in T_core_ after a 24 h period. A continuously lower T_core_ after an initial phase of 24 h may be explained by several candidates, such as hypoxic-induced hypometabolism [32–34], or nitric oxide pathway -involved mechanisms on thermoregulation [35, 36]. Moreover, effects of acute exposure to hypoxia on T_core_ has also been demonstrated to be strongly affected by T_a_ [19, 34]. Yet, it is still unclear why combined effects of cold, hypoxia, and dehydration had the most impact on the lowest AUC of T_core_ compared with other seven environmental conditions. One possible explanation is a loss of plasma volume with 48 h water deprivation before the main experiment. A previous study reported an initial increase in body surface temperature during hypoxia, and hence an initial increase in peripheral blood flow during hypoxia may indicate the shifting of heat away from the core to the periphery to facilitate cooling [34]. If this hypothesis were true despite dehydration status in this experiment, blood flow redistribution from core to peripheral tissues might dominate peripheral tissues to core transfer for protection in T_core_ decreases, and hence, T_core_ further decreases under combined conditions (cold, hypoxia, and dehydration). This hypothesis is speculative, and further experiments are needed.

Regarding to activity counts, significant main effects were found for oxygen and T_a_. Specifically, T_a_ of 24 °C or a hypoxic condition showed significantly lower values for activity counts. These results cincture with a previous study, showing activity counts decreased with T_a_ increase and an observation of hypoxic-induced lower locomotor activity counts [19]. Unexpectedly, none of the environmental conditions affect HLI. Some methodological consideration should occur. HLI was evaluated at the start and the end of the 24 h period, and therefore, continuous assessment of HLI will be investigated in future studies.

Our findings may be informative for populations who are working and performing in severe environmental conditions (i.e., cold and hypoxia), Specifically, humans cannot control environmental conditions; however, as dehydration status may cause further reductions in T_core_, an appropriate beverage intake could be effective tactics.

In summary, multiple environmental stressors (cold, hypoxia, and dehydration) have the most impact on the lower T_core_, as observed in the rats in this experiment. Moreover, locomotor activity counts and HLI do not affect this lower T_core_.

## Limitations

There are several limitations in the study. Firstly, the small size (n = 5 for each) should be re-considered. A *pos-hoc* power analysis for pairwise comparisons with significant difference (i.e., T_core_ AUC) was used as the standard of 80% power with a two-sided significance level of 0.05 (G Power 3.1). When considering each simple main effect of cold, hypoxia, or dehydration, a sample size of three in each group was necessary to achieve the appropriate statistical power for significant comparisons (Fig. 2b). However, more than 20 for each condition would have been necessary for other non-significant comparisons. Second, to clarify the underlying precise mechanisms of the lowest T_core_ with the combined effects, the metabolic rate (oxygen consumption), and HLI should have been measured continuously and other influencing factors on thermoregulation or body fluid regulatory hormones (e.g., thyroid hormone, noradrenaline, glucagon, renin, aldosterone, and vasopressin) should have been measured.

## Supplementary information

**Additional file 1.** **Supplemental table.** Body weight (BW) changes 48 hours before the experiment and just before the main experiment (24 h exposure) in each condition.

## Data Availability

The datasets used and/or analyzed during the current study are available from the corresponding author on reasonable request.

## Abbreviations

AUC: Area under the curve
HLI: Heat loss index
T_a_: Ambient temperature
T_core_: Core temperature
