# Supplementary material for: Combined stimuli of cold, hypoxia, and dehydration status on body temperature in rats: a pilot study with practical implications for humans
Source: BMC Res Notes. 2020 Nov 11;13:530. doi: 10.1186/s13104-020-05375-w (PMC7661168; doi:10.1186/s13104-020-05375-w)
Supplement: Supplementary file 1 — Additional file 1. Supplemental table. Body weight (BW) changes 48 hours before the experiment and just before the main experiment (24 h exposure) in each condition. [file 13104_2020_5375_MOESM1_ESM.docx]

**Supplemental Table** Body weight (BW) changes 48 hours before the experiment and just before the main experiment (24 h exposure) in each condition.

|  | BW at 48 h before (g) | | | BW just before (g) | | | % BW change | | |
| --- | --- | --- | --- | --- | --- | --- | --- | --- | --- |
| 24 °C + 21% O_2_ + Eu | 288.4 | ± | 13.5 | 295.2 | ± | 12.6 | 2.3 | ± | 0.5 |
| 10 °C + 21% O_2_ + Eu | 300.2 | ± | 30.8 | 310.4 | ± | 29.7 | 3.4 | ± | 1.1 |
| 24 °C + 21% O_2_ + De | 327.3 | ± | 25.7 | 298.0 | ± | 26.3 | -9.0 | ± | 1.0 |
| 24 °C + 12% O_2_ + Eu | 312.7 | ± | 25.5 | 322.3 | ± | 27.3 | 3.1 | ± | 0.9 |
| 10 °C + 12% O_2_ + Eu | 314.5 | ± | 35.8 | 324.1 | ± | 33.5 | 3.2 | ± | 1.4 |
| 24 °C + 12% O_2_ + De | 298.3 | ± | 30.8 | 272.9 | ± | 30.7 | -8.5 | ± | 2.0 |
| 10 °C + 21% O_2_ +De | 326.1 | ± | 42.4 | 296.5 | ± | 42.8 | -9.2 | ± | 1.2 |
| 10 °C + 12% O_2_ + De | 283.7 | ± | 53.9 | 255.3 | ± | 48.5 | -10.0 | ± | 2.6 |

Values are mean ± standard deviation. BW, body weight; Eu, euhydration; De, dehydration.
